# Comparison of platelet function between sedentary individuals and competitive athletes at rest

**DOI:** 10.1186/1477-9560-4-10

**Published:** 2006-08-17

**Authors:** Giuseppe Lippi, Martina Montagnana, Gian Luca Salvagno, Massimo Franchini, Gian Cesare Guidi

**Affiliations:** 1 Sezione di Chimica e Microscopia Clinica, Università degli Studi di Verona, Verona, Italy; 2 Servizio di Immunoematologia e Trasfusione, Azienda Ospedaliera di Verona, Verona, Italy

## Abstract

**Background:**

There are controversial evidences on the effect of different types and workloads of physical exercise on primary hemostasis. In particular, little is known on the chronic influence of a strenuous and regular aerobic training regimen on platelet function.

**Methods:**

The aim of this investigation was to compare platelet function between sedentary controls and trained athletes at rest and to evaluate whether a greater amount of exercise performed in professional cyclists may contribute to increased platelet chronic responsiveness compared to both elite cyclists and sedentary individuals. Platelet's ability to adhere and aggregate was assayed following a 12–24 h resting period in 49 active professional male road cyclists, 40 elite male cyclists and 43 matched sedentary healthy male volunteers, by the platelet function analyzer 100 (PFA-100).

**Results and discussion:**

Mean values of the collagen-epinephrine test did not differ between controls and athletes (sedentary controls: 111 ± 33 s; elite athletes: 113 ± 26 s, p = 0.93; professional athletes: 120 ± 33 s; p = 0.33), whereas mean values of the collagen-ADP test displayed a slightly but significant trend towards decreased values when comparing sedentary controls (83 ± 21 s) with either elite (77 ± 11 s, p < 0.01) or professional (75 ± 16 s, p < 0.01) athletes.

**Conclusion:**

The trend towards slightly lower collagen-ADP values are suggestive for a modest but significant chronic activation of primary hemostasis, highlighting the need to set appropriate reference ranges for the PFA-100 when evaluating primary hemostasis in physically active subjects.

## Background

Recent guidelines recommend 30 minutes or more of daily, moderate-intensity physical exercise on the basis of favorable biological adaptations and substantial health benefits, which include a reduced risk of developing cardiovascular disorders, cancer and other chronic pathologies [1]. Although it appears to exist a linear relation between physical activity and global health status, such that a further increase in physical activity and fitness will lead to additional improvements in health status [2], there is debate regarding intensity and type of physical activity required to achieve beneficial health changes without overwhelming favorable outcomes and eliciting cardiovascular abnormalities not present at rest [1,3]. In particular, there are still controversial evidences on the relation between physical exercise and primary hemostasis [3-10]. Therefore, the aim of this investigation was to compare platelet function between sedentary controls and trained athletes at rest and to evaluate whether a greater amount of exercise performed in professional cyclists may contribute to influence the chronic platelet responsiveness compared to both elite cyclists and sedentary individuals.

## Methods

Platelet's ability to adhere and aggregate was monitored with the use of the platelet function analyzer 100 (PFA-100, Dade International Inc. Miami, FL, USA) PFA-100 in 49 active professional male road cyclists, 40 elite male cyclists and 43 sedentary healthy male volunteers, matched for ethnic origin (all Caucasian), age (sedentary controls: 29 ± 9 y, elite cyclists: 28 ± 12 y, *p *= 0.501; professional cyclists: 28 ± 4 y, *p *= 0.311) and smoking habit (all non-smokers). Unlike platelet aggregation studies, the PFA-100 is a test relatively easy and rapid to perform, which provides a rapid evaluation of primary hemostasis and platelet function on whole blood anticoagulated specimens. The analyzer uses a disposable cartridge which internal active membrane is coated with either collagen-ADP (CADP) or collagen-epinephrine (CEPI). The interaction of whole anticoagulated blood with the agonists, in condition of high shear rates, triggers platelet activation and the resulting formation of a stable platelet plug interrupts the blood flow, which is recorded in term of closure time (CT) [11]. Athletes reached our Center in the middle of the competitive season, during a regular, high-workload period of aerobic training. All subjects recruited to the study were in a fasted state and athletes had rested for a period of 12–24 h since the last training session or competition. The consumption of drugs known to interfere with platelet function, along with clinically symptomatic platelet disorders, were carefully excluded by clinical history and physical examination in both the athlete and control populations. The research was carried out according to the principles of the Declaration of Helsinki; the study protocol was reviewed and approved by the ethics committee of our Department, and informed consent for testing was received from all individuals included in this study. Blood was collected in the morning from fasting subjects by venipuncture with 20 gauge (G) straight needles (Terumo Europe NV, Leuven, Belgium), directly into siliconized vacuum tubes containing 0.105 mol/l buffered trisodium citrate (Becton-Dickinson, Oxford, UK). Samples for PFA-100 testing were immediately analyzed, whereas specimens for coagulation studies were centrifuged at 1500 g for 10 min at 15°C, plasma was separated, stored in aliquots and kept frozen at -70°C until measurement. Von Willebrand factor (VWF) was measured on the Mini Vidas Immunoanalyzer (bioMerieux, Marcy l'Etoile, France), by a rapid and quantitative enzyme-linked immunosorbent assay. Significance of differences between samples was assessed by Student's t-test; the level of statistical significance was set at *p *< 0.05.

## Results and discussion

**Figure 1:**
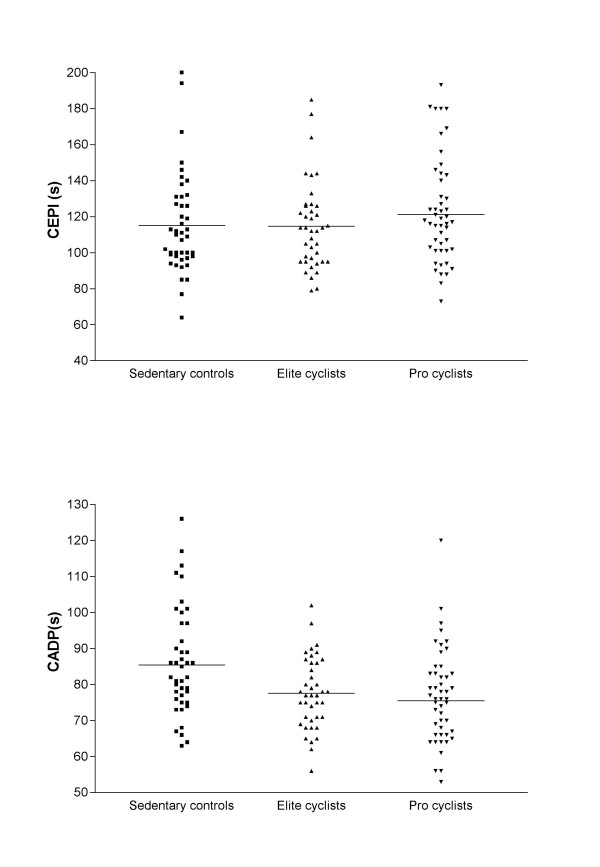
Value distribution of collagen-ADP (CADP) or collagen-epinephrine (CEPI) induced aggregation on the PFA-100 system in sedentary healthy controls, elite and professional road cyclists. The central horizontal line indicates the mean value.

The average intensity of daily physical exercise was substantially different among the three study populations (sedentary controls <10 min/day, elite cyclists 30–90 min/day and pro cyclists >90 min/day). When comparing results of the PFA-100 between sedentary individuals (CADP: 83 ± 21 s; CEPI: 111 ± 33 s) and athletes, mean values of CEPI appeared substantially unvaried by the physical activity (elite athletes: 113 ± 26 s; *p *= 0.93 and professional athletes: 120 ± 33 s; *p *= 0.33), whereas mean CADP values displayed a slightly but significant trend towards decreased values in either elite (77 ± 11 s; *p *< 0.01) or professional (75 ± 16 s; *p *< 0.01) athletes (Figure 1). In athletes, and particularly in professionals, the distribution is more homogeneous for CAPD values than CEPI ones, regardless of a similar number of outliers for both CEPI and CAPD, which are correspondent to the same athletes. Results of VWF measurement did not differ significantly between controls (106 ± 14 IU/dl) and either elite (107 ± 13 IU/dl, *p *= 0.258) or professional cyclists (108 ± 14 IU/dl, *p *= 0.144).

Growing evidences indicate that benefit of physical activity on cardiovascular disease may result to some extent from effects on hemostasis [10-13]. Accordingly, for rehabilitation training it is recommended that the intensity of exercise should be distinctly below the individual anaerobic threshold. It is hence debated whether a strenuous aerobic exercise might counterbalance these advantageous modifications [1-3] and no definitive data are still available on the influence of physical exercise on primary hemostasis. Although it has been reported that acute bout of moderate-intensity exercise may reduce platelet activation [4], earlier studies observed acute platelet activation, aggregation and adhesion during physical exercise, as evaluated by ADP and collagen-induced aggregation [5], platelet granularity [6], filtragometry [7], enhanced release of soluble P-selectin and intracellular Ca^2+ ^[8], downregulation of glycoprotein Ib [9] and moderate increase of platelet-leukocyte conjugates [10].

However, no data are available so far for the PFA-100 analyzer on blood samples collected from professional athletes at rest, undergoing a strenuous and regular aerobic training regimen. Although this is only a preliminary observation and even though the values of the PFA-100 observed in competitive athletes are still within normal ranges [14], the modestly decreased CADP values provide evidence of a slight, persistent platelet activation in elite and professional athletes, providing further insight to the controversy whether an increased physical activity may chronically influence hemostasis. In this regard, we conclude that gradually increased aerobic training regimens may produce a modest but significant chronic activation of primary hemostasis.

At variance with earlier studies, the innovative value of this investigation is represented by the original study design, involving a large subset of elite and professional endurance athletes who were tested following a 12–24 h resting period. Unfortunately, there is no definitive explanation to justify the discrepant behavior between CADP and CEPI in athletes. The activation of platelet adhesion/aggregation can be induced by means of heavy exercise, though warm-up exercise diminishes this effect in sedentary men [15]. Therefore, the continuous exercise, even of low intensity and possibly not reported by the athletes, may have contributed to the increased platelet responsiveness to CADP. Then, due to the inverse correlation between hematocrit and closure time under all circumstances, the hematocrit value is an additional variable that should be taken into consideration for the correct interpretation of PFA-100 measurements [16]. Accordingly, the different hematologic profile of competitive athletes [17] may provide a further explanation for the discrepancy observed between CADP and CEPI values in the athletes population.

## Conclusion

The influence of common variables influencing PFA-100 results such as acquired or congenital platelet disorders and VWF levels has been accurately excluded in our study populations. Therefore, the trend towards slightly lower CADP values in athletes is suggestive for a moderate chronic platelet activation and deserves further scrutiny, highlighting that appropriate reference ranges for the PFA-100 may be implemented when evaluating primary hemostasis in physically active subjects.

## Authors' contributions

GL: conceived of the study, participated in its design and coordination and drafted the manuscript; MM: participated in the design of the study, performed the statistical analysis and helped to draft the manuscript; GLS: recruited the study population, collected the samples and performed the laboratory testing; MF: recruited the study population and participated in the design of the study, GCG: participated in the design and coordination of the study.
